# Methods of deliberate self-harm in a tertiary hospital in South Africa

**DOI:** 10.4102/sajpsychiatry.v26i0.1399

**Published:** 2020-04-21

**Authors:** Deirdre Pieterse, Jacqueline Hoare, Kerry-Ann Louw, Elsie Breet, Michelle Henry, Ian Lewis, Jason Bantjes

**Affiliations:** 1 Department of Psychiatry and Mental Health, University of Cape Town, Cape Town, South Africa; 2 Department of Psychiatry, Faculty of Health Sciences, Stellenbosch University, Cape Town, South Africa; 3 Department of Psychology, Stellenbosch University, Cape Town, South Africa; 4 Centre for Higher Education Development, University of Cape Town, Cape Town, South Africa

**Keywords:** deliberate self-harm, non-fatal suicide, general hospital, mental health, methods of self-harm, means restriction, suicide prevention

## Abstract

**Background:**

Little is known about the methods of deliberate self-harm (DSH) in South Africa (SA), despite the importance of means restriction as a public health strategy to reduce the morbidity and mortality associated with self-harm.

**Aim:**

The aim of this study was to investigate the range of methods used in DSH and identify the socio-demographic and clinical factors associated with violent and non-violent methods of DSH among patients treated at a tertiary hospital in SA.

**Setting:**

The study was conducted at an urban, tertiary level emergency department at Groote Schuur hospital in Cape Town, South Africa.

**Method:**

Data were collected from 238 consecutive DSH patients who presented for emergency department treatment at the hospital. Logistic regression models were used to explore the factors associated with violent and non-violent methods of DSH.

**Results:**

Self-poisoning was the most common method of self-harm (80.3%). Prescription medication was the most common form of self-poison (57.6%), while a large number of patients used non-prescription paracetamol (40.9%). In the regression analysis, male gender, stating that the reason for DSH was to escape a situation and history of substance use were associated with violent method of DSH.

**Conclusion:**

Improved monitoring of prescription medications commonly used in DSH is integral to public health suicide prevention strategies in SA. This study underscores the need for substance use interventions in the healthcare setting.

## Introduction

In South Africa (SA), suicide is a serious public health concern^1^ accounting for approximately 9.6% of unnatural deaths and yielding an annual prevalence rate of 13.25 per 100 000.^2^ Studies in this country have consistently shown that common methods of suicide are hanging, firearms and poisoning.^2,3,4^ Globally, 30% of all fatal suicide attempts are because of pesticide self-poisoning, occurring most frequently in rural agricultural areas.^5^

Researchers recognise the confusing and often contradictory definitions of suicide and suicide behaviour in the literature.^6^ Inadequate definitions in suicide research often limit the generalisability of findings. The term suicidal behaviour has a broader meaning than suicide and encompasses a range of emotions, cognitions and actions, all of which are characterised by a conscious desire to die.^2^ A distinction is also made between ‘deliberate self-harm (DSH) with intent to die’ (i.e. attempted suicide) and other forms of repetitive self-harm (such as cutting, self-mutilation and hitting) which is self-inflicted, habitual and is carried out without any conscious intention to die. Deliberate self-harm with intent to die is a form of suicidal behaviour. ‘Deliberate self-harm’ and ‘non-fatal suicide’ are also referred to as ‘suicide attempts’ in the literature. The focus of this study is DSH regardless of intent to die.

In the last 15 years, a number of descriptive general hospital-based studies have investigated the epidemiology of DSH in urban and rural hospitals in SA.^7,8,9,10,11,12,13,14^ The methodological differences across these studies make it difficult to generalise findings; however, data suggest that self-poisoning is the most common method of DSH in SA.

Self-poisoning was found to be the most common method of DSH in international studies conducted in hospital emergency departments (EDs).^15,16,17,18,19^ Prescription medication was the most frequently used substance in the cited studies in high-income countries, with the exception of the United Kingdom where paracetamol was the most common medication used in DSH.^19^ Although the correlates and risk factors of DSH have been described in international literature, the factors associated with methods of DSH have not been extensively examined in SA.

Evidence suggests that limiting access to lethal means (‘means restriction’) is effective in reducing morbidity and mortality.^20^ Furthermore, the World Health Organization (WHO) states that knowledge of the most commonly used suicide methods in a sub-population is important to implement specific suicide prevention strategies.^5^

It is within this context that we set out to investigate the methods of DSH and the socio-demographic and clinical correlates of these methods among patients presenting to a tertiary hospital in SA.

## Methods

This descriptive, cross-sectional study was undertaken in the ED of an urban, tertiary hospital in SA. The study hospital is an 893-bed, government-funded hospital^21^ serving an estimated 1.7 million people.^22^ The majority of patients are referred from a regional or district hospital if they require tertiary care, meaning that the hospital manages more complex patients. The hospital has a 24-h psychiatry service and 40 inpatient beds dedicated to psychiatry supported by four psychiatrists. The hospital serves patients older than 12 years.

Deliberate self-harm was defined using the WHO or Euro Multicentre intervention study on parasuicide definition:

[*A*]n act with non-fatal outcome, in which an individual deliberately initiates a non-habitual behaviour that, without intervention from others, will cause self-harm, or deliberately ingests a substance in excess of the prescribed or generally recognised therapeutic dosage, and which is aimed at realising changes which the subject desired via the actual or expected physical consequences.^23^

All DSH patients, regardless of the intention of the self-harm, who presented to the ED following an act of DSH were included. The study period was from the 16 June 2014 to March 2015. Data were collected by a registered psychiatric nurse with more than 20 years of experience in emergency psychiatry using a data capture form which was piloted prior to the commencement of the study. Data extraction was reviewed by a PhD candidate in psychology to ensure that information had been correctly captured from patient records. The presence or absence of a psychiatric diagnosis was assessed from the clinical notes in patients’ folders. The study did not use structured clinical interviews to determine a clinical diagnosis and relied on folder diagnoses.

Over this time, there were 270 presentations of DSH of whom 32 were excluded. The exclusions were for the following reasons: death as a result of DSH (five patients), patients who left the hospital prior to data capture,^1^ missing folders^17^ and patients presenting with DSH for the second time during the study period.^9^

### Measures

The following data were collected from clinical folders and possibly supplemented with patient interview:

- *Socio-demographic information.* Self-reports of patients’ age, gender, race, relationship status and employment status were recorded on a data capture form.
- *Method of self-harm*. The method of non-fatal self-harm was recorded. A distinction between the use of prescription and non-prescription medications was recorded based on the following definition: prescription medication was defined using the South African Medicines Control Council definition as ‘a substance which can only be obtained on the prescription of an authorised prescriber’.^24^ Violent (i.e. those which entailed damage to bodily tissue) and non-violent methods (self-poison) were also recorded.
- *Details of self-harm.* Self-report data were obtained about reasons given for self-harm and the patient’s stated intention for engaging in the behaviour.
- *Clinical characteristics and medical interventions.* Information about the level of consciousness on admission (as measured by the Glasgow Coma Scale), psychiatric history, history of substance use and current psychiatric diagnosis were obtained from the patients’ files. We also recorded the details of the hospital-based management, including the level of admission and the interventions provided. Level of medical admission was defined by the following categories: admission to intensive care unit (ICU), admission to long-stay medical or surgical ward, admission to short-stay medical ward, admission to emergency psychiatric ward and total number of days spent in hospital. Medical intervention and assessment was defined by whether or not a patient received a medical intervention, assessment by psychologist or psychiatric assessment.

### Data analysis

Descriptive statistics were used to provide epidemiological information about the methods of DSH and the sample characteristics. For the purposes of statistical analysis, we differentiated between violent (damage to bodily tissue) and non-violent (self-poison) methods. Chi-square tests of association were conducted to determine which socio-demographic and clinical variables were associated with the specified method of DSH.

The variables found to be significantly associated with the method of DSH in bivariate analysis were entered into the logistic regression models. Logistic regression analysis was used to determine which factors (demographic and clinical variables) were associated with the use of a violent method and non-violent method of DSH. All analyses were conducted using SPSS software (version 24) and the significance level was set at *α* = 0.05.

### Ethical considerations

Ethical approval for this study was obtained from the University Human Research Ethics Committee (HREC/REF 687/2016). Institutional permission to conduct this study in the hospital was obtained from both the Department of Health and the relevant hospital authorities.

## Results

### Socio-demographic and clinical characteristics of the sample

The description of the sample is shown in Table 1. The mean age of the sample was 31.5 years (SD = 13.9, range = 13–82). More than a third (37.4%) of the total sample reported previous suicide attempts.

**TABLE 1:** Description of sample and method of self-harm (*N* = 238).

Socio-demographic and clinical description of the sample	*n*	%
**Gender**		
Male	96	40.3
Female	142	59.7
**Race**		
Black	82	34.5
Asian	8	3.4
Mixed race	103	43.3
White	33	13.9
Not known	12	5.0
**Home language**		
Afrikaans	49	20.6
English	135	56.7
isiXhosa	47	19.7
isiZulu	2	0.8
Not known	5	2.1
**Relationship status**		
Married	42	17.6
In a relationship	4	1.7
Single	171	71.8
Divorced	14	5.9
Widowed	6	2.5
Not known	1	0.4
**Number of dependents**		
No dependants	155	65.1
Dependants	80	33.6
Not known	43	1.3
**Completed level of education**		
Primary school	100	42.0
Secondary school	100	42.0
Tertiary education	38	16.0
**Employment status**		
Employed	51	21.4
Student	46	19.3
Unemployed	130	54.6
Retired	6	2.5
Not known	5	2.1
**Socio-economic status**		
Low-to-moderate income (R0–R76 800)^1^	131	55.0
High income (R76 801–R2 457 601)	85	35.7
Not known	22	9.2
**Current psychiatric diagnosis**		
Yes	143	60.1
No	66	27.7
Not known	29	12.2
**History of previous suicidal self-injury**		
Previous attempt	89	37.4
No previous attempt	69	29.0
Not known	80	33.6

Note: As per Statistics South Africa Census 2011.

### Methods of self-harm

The methods of DSH are shown in Table 2. Self-poisoning was the most common method reported in this sample (80.3%). The range of medications used in self-poisoning is shown in Table 3. Among patients who reported self-poisoning, prescription medications were most commonly used (57.6%). Of the prescription medication, other medication unknown or not specified by the patient (23.5%), tricyclic antidepressants (12.7%), anti-hypertensive agents (12.7%) and benzodiazepines (10.9%) were most commonly used.

**TABLE 2:** Method of deliberate self-harm.

Method of deliberate self-harm	*n*	%
**Self-poison**	191	80.3
Prescription medication only	79	33.2
Non-prescription medication only	38	16.0
Ingestion or inhalation of poison	19	7.9
Prescription + non-prescription medication	49	20.6
Prescription medication + ingestion/inhalation	1	0.4
Non-prescription medication + ingestion/inhalation	4	1.7
All three self-poison methods	1	0.4
**Damage bodily tissue**	34	14.3
Laceration	13	5.5
Hanging	13	5.5
Laceration + hanging	1	0.4
Asphyxiation	0	0.0
Immolation	0	0.0
Jumped off a height	4	1.7
Jumped in front of train	3	1.3
**Self-poison and damage bodily tissue**	8	3.3
**Not known**	5	2.1

**TABLE 3:** Prescription and non-prescription medication taken by patients.

Medication type	*n*	%
**Prescription**	221	**-**
Other prescription medication unknown or not specified by the patient benzodiazepines	52	23.5
Tricyclic antidepressants	28	12.7
Anti-hypertensives	28	12.7
Benzodiazepines	25	11.3
Analgesic medication	23	10.4
SSRI	18	8.1
Anti-epileptic medication	14	6.3
Anti-psychotics	13	5.9
Oral hypoglycaemic agents	10	4.5
Antibiotics	10	4.5
**Non-prescription**	141	-
Paracetamol	54	38.3
Other medication unknown or not specified by the patient	18	12.8
Antihistamine	17	12.0
NSAID	12	8.5
Paracetamol and codeine prep	10	7.1
Vitamin compound	10	7.1
Iron tablets	9	6.4
Aspirin	6	4.2
Illicit substance	5	3.5

SSRI, selective serotonin reuptake inhibitor; NSAID, non-steroidal anti-inflammatory drug.

Paracetamol (40.9%), anti-histamines (12.9%), other non-prescription medication unknown or not specified by the patient (12.8%) and non-steroidal anti-inflammatory drugs (9.1%) were the most common non-prescription medications used to self-harm. Nineteen (7.9%) patients reported ingestion or inhalation of poison. Notably, 20% of patients used both prescription and non-prescription medication, while only one patient used all three self-poison methods. Thirty-four patients (14.3%) used damage to bodily tissue as a method of DSH. Attempted hanging and laceration to the skin were the most common violent methods of DSH (5.5%, *n* = 13).

### Substance use

A total of 20.2% (*n* = 48) patients reported single substance use at the time of the attempt, while 18% reported polysubstance use. Alcohol (65.5%, *n* = 38 instances of alcohol use) was the most commonly used substance, while methamphetamine (13.8%, *n* = 8), cocaine (6.9%, *n* = 4), cannabis (5.2%, *n* = 3), heroin (5.2%, *n* = 3), methylenedioxymethamphetamine (ecstasy) and opiates (1.7%, *n* = 1 each) use were also reported. A total of 37.4% (*n* = 89) of patients reported a history of a substance use disorder.

### Stated intention and reason for self-harm

The degree of intent was reported as follows: 66% did not express an intention to die, while 34% indicated an intent to die. Impulsivity was reported in 22.3% of the sample, while a majority (87.8%) reported that the self-harm attempt was non-accidental. Among the total sample, the most common intentions were to communicate something, for example, distress (34.5%, *n* = 82), to die (34%, *n* = 81), to regulate the behaviour of someone else (23.1%, *n*= 5 5), an impulsive act (22.3%, *n* = 53) or to escape a situation (20.6%, *n* = 49). Family conflict (36.6%) and relationship (friendship, marital or romantic) issues (31.1%) were the most common stated reasons for DSH.

Other stated reasons were psychiatric illness (15.1%) and financial concerns (19.7%).

### Severity of injuries and clinical management of patients

Of the total sample, 15.5% were assessed as having a moderate-to-severe brain injury (Glasgow Coma Scale < 13) upon arrival at the hospital. Two-thirds of the sample received a medical intervention, and in 71.8% of the sample, psychotropic medication was initiated. Of the total sample, 84 (35%) patients were seen in the ED and discharged, and 90 (38%) were admitted to an emergency psychiatric unit (EPU). The 150 patients who were admitted spent a combined total of 1186 days in hospital (mean = 6.71 days, standard deviation [SD] = 9.31).

### Correlates of violent and non-violent methods of self-harm

The results of the unadjusted and adjusted regression coefficients of tests of association with methods of DSH are shown in Table 4. Age was analysed as a continuous variable. The mean age of those who used violent methods was 33.62 years (SD 13.36) and for those who used non-violent methods it was 31.28 years (SD 14.25) (*t* = −0.89; *p* = 0.375). Socio-demographic and clinical variables identified as significant in the univariate analyses were entered into a logistic regression model. The regression model was significant, *χ*^2^(5) = 42.938, *p* < 0.001, Nagelkerke *R*^2^ = 0.335. This model accurately predicted use of violent or non-violent DSH method 87% of the time.

**TABLE 4:** The unadjusted and final regression coefficients form the bivariate and motivate analysis of factors associated with method of self-harm.

Predictors of DSH method	Unadjusted			Adjusted			
	*χ*^2^	*p*	*V*	Wald statistic	Adjusted odds ratio	*p*	95% CI
**Socio-demographic factors**							
Male gender	28.972	< 0.001	0.356	14.687	6.198	< 0.001	2.438–15.755
Race	5.275	0.153	0.156	-	-	-	-
Relationship status	0.057	0.822	0.016	-	-	-	-
Number of dependants	1.569	0.210	0.083	-	-	-	-
Completed level of education	0.465	0.793	0.045	-	-	-	-
Employment status	2.594	0.459	0.108	-	-	-	-
Socio-economic status	1.750	0.186	0.092	-	-	-	-
**Clinical factors**							
History of psychiatric diagnosis	2.756	0.097	0.117	-	-	-	-
Current psychotic disorder	23.157	**< 0.001**	0.339	-	-	-	-
Current MDD	2.262	0.133	0.106	-	-	-	-
Current adjustment disorder	0.210	0.647	0.032	-	-	-	-
Current PTSD	0.394	0.530	0.044	-	-	-	-
Current substance use disorder	3.776	0.052	0.137	-	-	-	-
Current anxiety disorder	0.524	0.469	0.051	-	-	-	-
Current bipolar disorder	0.394	0.530	0.044	-	-	-	-
Current personality disorder	1.507	0.220	0.086	-	-	-	-
History of previous DSH	0.444	0.505	0.054	-	-	-	-
History of substance abuse	20.857	**< 0.001**	0.302	-	-	-	-
Substance use during DSH	1.527	0.217	0.085	7.710	3.288	0.005	1.419–7.617
**Stated intention for DSH**							
To escape a situation	4.037	0.045	0.133	6.394	3.432	0.011	1.320–8.926
To die	0.097	0.756	0.021	-	-	-	-
To regulate the behaviour of someone else	2.795	0.095	0.111	-	-	-	-
To regulate own emotional state	0.335	0.563	0.038	-	-	-	-
To communicate something	0.385	0.535	0.041	-	-	-	-
Implosive act (anger)	1.137	0.286	0.071	-	-	-	-
Mistake	0.000	1	0.000	-	-	-	-
Impulsive act	0.608	0.436	0.052	-	-	-	-
Intention unknown	0.408	0.523	0.042	-	-	-	-
Other intention	1.614	0.204	0.084	-	-	-	-
**Stated reason for DSH**							
Psychiatric illness	9.983	0.002	0.209	-	-	-	-
Financial concerns	0.050	0.823	0.015	-	-	-	-
Relationship issue	1.996	0.158	0.094	-	-	-	-
Family conflict	1.844	0.175	0.090	-	-	-	-
Medical illness	0.054	0.817	0.015	-	-	-	-
Bereavement	0.084	0.772	0.019	-	-	-	-
Academic concerns	0.128	0.720	0.024	-	-	-	-
Social issues	0.478	0.490	0.046	-	-	-	-
Unplanned pregnancy	0.608	0.436	0.052	-	-	-	-
Mistake	0.608	0.436	0.052	-	-	-	-
Reason unknown	1.341	0.247	0.077	-	-	-	-
Other reason	0.388	0.533	0.041	-	-	-	-
Constant	-	-	-	56.290	0.027	< 0.001	-

DSH, deliberate self-harm; MDD, major depressive disorder; PTSD, post-traumatic stress disorder.

However, in the final step of the regression model, the following variables were no longer significant: patients who stated that their psychiatric illness was the reason for DSH (*p* = 0.279) and patients with a current diagnosis of a psychotic disorder (*p* = 0.100). These variables were therefore removed from the model. The new final regression was significant, *χ*^2^(3) = 38.348, *p* < 0.001, with Nagelkerke *R*^2^ = 274. This model accurately predicted use of violent or non-violent DSH method 87% of the time. Male gender, wanting to escape a situation, and a history of substance abuse meant it more likely for someone to use a violent method of DSH. Men were 6.2 times more likely to use a violent method of DSH compared with women. Patients whose intention was to escape a situation were 3.4 times more likely to use a violent method of DSH compared with patients who expressed other intentions. Finally, patients with a history of substance abuse were 3.3 times more likely to use a violent method of DSH compared with patients with no prior history of substance abuse.

## Discussion

Self-poison was the most common method of DSH seen at the hospital over the study period. Prescription medication was the most prevalent form of self-poison, while a significant number of patients used the non-prescription medication paracetamol. This study provides detail on violent methods of DSH in this sample population. In the regression analysis, male gender, stating that the reason for DSH was to escape a situation and history of substance use were associated with violent method of DSH.

The main finding is consistent with the literature: self-poison with possible fatal consequences remains the most common form of DSH worldwide.^7,10,16,19^ Despite the known worldwide burden of self-poisoning and DSH, few standard hospital-based, mental health intervention protocols exist to manage patients who present with DSH in the ED.^25^

The prevalence of prescription medication in this sample further supports the idea that ingestion of agricultural poison as a method of DSH is less common in urban areas of SA.^7,11^ Benzodiazepines and anti-depressants are consistently reported as highly prevalent substances used in DSH worldwide.^16,19,26^ This study replicates this finding in SA and it has important implications for public health intervention regarding medicine control. In this study, tricyclic antidepressants, anti-hypertensive agents and benzodiazepines were the most frequently used medications, which correlates the findings of Raubenheimer and Jenkins.^11^

Paracetamol remains readily available in unlimited quantities in supermarkets and pharmacies in SA, and consistent with local studies, a significant number of patients used paracetamol as a method of DSH.^7,8^ In the United Kingdom, a decrease in the frequency of paracetamol overdoses was observed when the tablets became available exclusively in blister pack form.^27^ As most acts of DSH are impulsive, it is proposed that limiting the quantity of tablets and increasing the time needed to ingest a toxic dose may allow time for reflection. This method of ‘means restriction’ has now become a recognised strategy for suicide prevention globally.^28^

Violent methods of DSH have largely been under-reported in hospital-based studies in SA. Congruent with data showing that hanging is the most prevalent method of fatal suicide in SA,^2^ attempted hanging was one of the most common methods of violent DSH seen in this sample. Our study found no incidents involving firearms and a relatively small number of patients who jumped from a height. This result may be explained by the high mortality associated with these methods. Although only 11% of patients in our study used both violent and non-violent methods of DSH, Haw et al.^29^ reported that this group has a higher suicidal intent and clinicians should be mindful of this when planning care and follow-up of this sub-group of patients.

The gender difference in choice of DSH method is in keeping with the SA data on fatal suicides. Men tend to use more violent methods of suicide such as hanging, while women are more likely to choose self-poisoning.^30^ In a study based in the United Kingdom, violent method of DSH was associated with high suicidal intent^29^ and researchers from both the USA and Norway have reported that patients who used a violent method are more likely to attempt and complete suicide in future.^31,32^ The authors found no significant associations between method of DSH and other socio-demographic factors such as age, employment or socio-demographic status.

This research finding that history of substance use is associated with violent method of DSH adds to the literature on the strong relationship between substance use and subsequent DSH. Harmful use of alcohol is historically a well-known risk factor for DSH and recent research continues to confirm this association in both hospital- and general population-based studies.^33,34,35^

Previous hospital-based research in SA has identified relationship difficulties, financial distress and distress at home as common reasons for DSH.^7,11,12^ In this study patients who stated that they wanted to escape a situation were more likely to use a violent method of DSH. This finding adds to the growing body of literature exploring the reasons for and correlates of DSH.

patients who stated that they wanted to escape a situation were more likely to use a violent method of DSH add to this growing body of the literature exploring the reasons for and correlates of DSH.

This study has several limitations. Firstly, the study was conducted in one tertiary hospital in the Western Cape which limits the generalisability of our findings. However, this study adds to the literature on methods used in DSH in SA and hospitals provide a unique opportunity to study this high-risk and understudied population. Secondly, we did not gather information on the source of the prescription medication used in DSH.

This information could be valuable to guide public health interventions and make recommendations to the local Medicines Control Council. Thirdly, personality profiles of the participants were not assessed. Finally, the cross-sectional design of the study limits our ability to comment on the direction of the associations in the regression analysis.

## Conclusion

This study contributes to emerging literature on methods used in DSH in SA. There is a need for the continual monitoring of methods of DSH and the collection of accurate and current epidemiological data about self-harm in SA. The population of DSH patients is continually evolving and the methods of self-harm change in response to dynamic factors, including the availability of means. It is of concern that self-poison and specifically prescription medication were highly prevalent methods of DSH in this population. There is an urgent need for more research on the source of prescription medication and its relationship to DSH. Limiting the quantity and reviewing the packaging of paracetamol available in supermarkets are effective strategies of means restriction that should be adopted in SA.
